# Quantifying motor–cognitive reserve using a novel multi-modal stress test

**DOI:** 10.1093/braincomms/fcaf412

**Published:** 2025-10-24

**Authors:** Tal Kozlovski, Inbal Maidan, Eran Gazit, Amgad Droby, Avner Thaler, Jeffrey M Hausdorff, Nir Giladi, Yoav Benjamini, Anat Mirelman

**Affiliations:** Laboratory of Early Markers of Neurodegeneration, Neurological Institute, Tel Aviv Sourasky Medical Center, Neurological Institute, Tel Aviv 6423906, Israel; Department of Statistic and Operations Research, School of Mathematical Sciences, Tel Aviv University, Tel Aviv 6997801, Israel; Laboratory of Early Markers of Neurodegeneration, Neurological Institute, Tel Aviv Sourasky Medical Center, Neurological Institute, Tel Aviv 6423906, Israel; Gray Faculty of Medicine and Health Sciences, Tel Aviv University, Tel Aviv 6997801, Israel; Sagol School of Neuroscience, Tel Aviv University, Tel Aviv 6997801, Israel; Center of the Study of Movement, Cognition, and Mobility, Neurological Institute, Tel Aviv Sourasky Medical Center, Tel Aviv 6423906, Israel; Laboratory of Early Markers of Neurodegeneration, Neurological Institute, Tel Aviv Sourasky Medical Center, Neurological Institute, Tel Aviv 6423906, Israel; Gray Faculty of Medicine and Health Sciences, Tel Aviv University, Tel Aviv 6997801, Israel; Sagol School of Neuroscience, Tel Aviv University, Tel Aviv 6997801, Israel; Laboratory of Early Markers of Neurodegeneration, Neurological Institute, Tel Aviv Sourasky Medical Center, Neurological Institute, Tel Aviv 6423906, Israel; Gray Faculty of Medicine and Health Sciences, Tel Aviv University, Tel Aviv 6997801, Israel; Sagol School of Neuroscience, Tel Aviv University, Tel Aviv 6997801, Israel; Gray Faculty of Medicine and Health Sciences, Tel Aviv University, Tel Aviv 6997801, Israel; Sagol School of Neuroscience, Tel Aviv University, Tel Aviv 6997801, Israel; Center of the Study of Movement, Cognition, and Mobility, Neurological Institute, Tel Aviv Sourasky Medical Center, Tel Aviv 6423906, Israel; Rush Alzheimer’s Disease Center and Department of Orthopaedic Surgery, Rush University Medical Center, Chicago, IL 60612, USA; Department of Physical Therapy, Gray Faculty of Medicine and Health Sciences, Tel Aviv University, Tel Aviv 6997801, Israel; Laboratory of Early Markers of Neurodegeneration, Neurological Institute, Tel Aviv Sourasky Medical Center, Neurological Institute, Tel Aviv 6423906, Israel; Gray Faculty of Medicine and Health Sciences, Tel Aviv University, Tel Aviv 6997801, Israel; Sagol School of Neuroscience, Tel Aviv University, Tel Aviv 6997801, Israel; Center of the Study of Movement, Cognition, and Mobility, Neurological Institute, Tel Aviv Sourasky Medical Center, Tel Aviv 6423906, Israel; Department of Statistic and Operations Research, School of Mathematical Sciences, Tel Aviv University, Tel Aviv 6997801, Israel; Edmond J. Safra Center for Bioinformatics, Tel Aviv University, Tel Aviv 6997801, Israel; Laboratory of Early Markers of Neurodegeneration, Neurological Institute, Tel Aviv Sourasky Medical Center, Neurological Institute, Tel Aviv 6423906, Israel; Gray Faculty of Medicine and Health Sciences, Tel Aviv University, Tel Aviv 6997801, Israel; Sagol School of Neuroscience, Tel Aviv University, Tel Aviv 6997801, Israel; Center of the Study of Movement, Cognition, and Mobility, Neurological Institute, Tel Aviv Sourasky Medical Center, Tel Aviv 6423906, Israel

**Keywords:** motor reserve, cognitive reserve, neurodegeneration, stress test

## Abstract

Reserve is a physiological capacity used under demanding situations. The concept was developed to account for the discrepancy between pathology and clinical manifestation. In neuroscience, motor, brain and cognitive reserves are abstract measures, conceptually defined yet elusive to quantify. Reserve is indirectly assessed using proxies such as years of education and brain volume, limiting its utility. Moreover, the dichotomy in definitions of cognitive and motor reserves is artificial, as daily function requires an intricate network of connections between these domains. Here, we assessed the validity of a newly developed graded motor cognitive ‘stress test’ to quantify the combined motor and cognitive reserve (MCR). The study included *144* participants (ages between 18 and 85, 50% women) with a range of reserve capacities (i.e. healthy young and older adults and individuals with Parkinson's disease, Alzheimer’s disease, dementia with Lewy bodies and mild cognitive impairment). The assessment included walking on a treadmill while negotiating motor and cognitive challenges delivered using virtual reality. To establish an MCR index score, we used a semi-supervised machine learning algorithm. The model includes performance measures from completing the stress test and measures obtained from wearable sensors used during the test. Validation of the proposed MCR index was examined through: (i) model face validity–reflecting decline of performance as challenge increased; (ii) known-groups validity—classification of scores according to neurological status; (iii) construct validity (convergent)—association with common MCRs proxies as well as MRI-derived regional brain volumes. The model's face validity revealed decreased performance with increased motor and cognitive challenges (both domains *P* < 0.001). The index accurately discriminated between healthy controls and those diagnosed with neurological conditions with an area under the curve of *0.89 [95% CI: 0.79–0.99]* which was significantly higher than all other commonly used proxies. Statistically significant Spearman's *ρ* correlations were observed with all commonly used motor and cognitive proxies (0.56 ≤ *r* ≤ 0.79, after multiplicity correction all *P* < 0.05), reflecting construct validity. In addition, statistically significant correlations were observed between the MCR index and whole-brain grey matter and white matter volumes (*r* = 0.63 and 0.55), as well as the pre-defined left and right caudate nucleus (*r* = 0.56 and 0.68) and inferior-frontal gyrus (*r* = 0.47 and 0.58). This proof-of-concept study shows that the novel MCR index is valid, with high sensitivity to neurological deficits and is able to quantify reserve on an individual level. This new innovative tool can assist in screening for motor cognitive deficits and potentially, for predicting motor and cognitive decline associated with neurodegenerative disease.

## Teaser

Kozlovski *et al*. present a novel motor–cognitive stress test that yields a score index aimed at capturing motor–cognitive reserve capacity. The authors validated the index across three aspects: face validity, discriminant validity and construct validity, supporting its potential for identifying individuals at risk for neurodegenerative diseases.

## Graphical Abstract

**Graphical Abstract:**
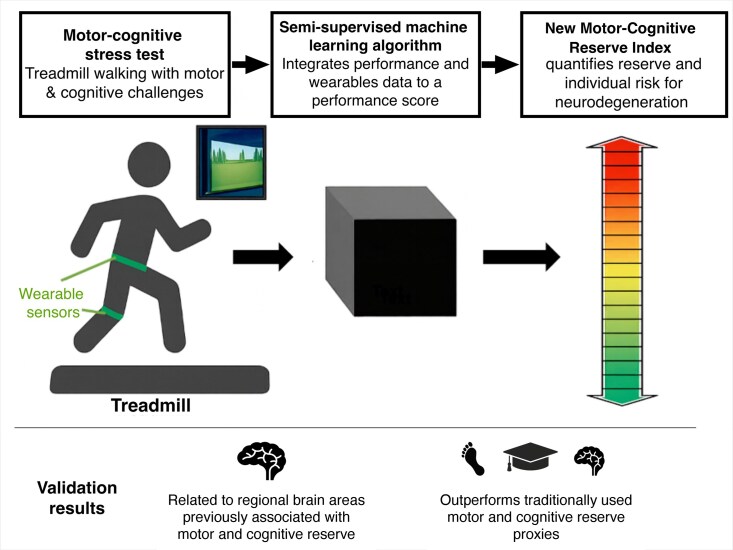

## Introduction

*Reserve* is a physiological capacity that may be utilized in demanding or challenging situations, extending beyond what is ordinarily used.^1^ In the medical field, this concept has been developed to account for the discrepancy between the primary pathological process and clinical manifestation. The concept of reserve is routinely used in several medical disciplines (e.g. cardiac,^2^ pulmonary,^3^ and renal reserve).^4^ In those disciplines, physiologic reserve was shown to have critical diagnostic and prognostic value. For example, reserve levels can dictate suitable treatment for renal transplanted patients.^5^ Physiologic reserve is generally assessed by subjecting the individual to a ‘stress test’, requiring the recruitment of additional resources to address the increasing physical or physiologic demands. In many instances, the increased challenge during the ‘stress test’ helps to unveil an underlying medical condition (e.g. cardiac changes).^6-8^

In neuroscience, the term reserve was first used in the context of cognitive decline among patients diagnosed with Alzheimer’s disease.^9-13^ In its current evolution, the term reserve includes two entities; cognitive and brain reserve. Reserve of either type represents individual variability in the functional use or structural integrity of the nervous system that alters a person’s cognitive and behavioural abilities following the onset of brain pathology. The former is considered adaptive and refers to efficiency, flexibility and resilience to pathology, while the latter relates to the structural components of the brain.^14^ In contrast, compensatory mechanisms refer to the brain's reactive adjustments—such as recruiting alternative networks—to cope with pathology after it has occurred. While conceptually related, cognitive reserve is typically seen as a *pre-existing* buffer, whereas compensation reflects *dynamic reorganization*.^15^ While the concept of reserve was introduced many years ago, the academic discussion on refining those definitions is still ongoing.^14,16,17^ Currently, there is no direct measurement for cognitive reserve; rather, it is assessed through proxies—measures found to correlate with cognitive decline—such as years of education, active lifestyle, occupational attainment and intelligence quotient (IQ)^18-25^ or calculated residual volumes.^26^

In recent years, there has been a growing body of work on motor reserve. This concept was applied to account for the variance between patients diagnosed with Parkinson’s disease with similar levels of pathology but different symptomatic presentation.^27,28^ Motor reserve, similar to cognitive reserve, is described using proxies—such as preferred walking speed,^29^ and a physically active lifestyle.^30,31^ Similar to cognitive reserve, direct measures of motor reserve are currently lacking.

Yet, the isolated dichotomous concepts of cognitive and motor reserves are somewhat artificial as many functions in daily life involve the utilization of multiple systems simultaneously, working as a network.^28,29,32-35^ MRI studies that explored associations between structural brain areas and motor or cognitive reserve showed positive correlations in total brain volume, head circumference,^36^ and caudate nucleus,^37,38^ putamen,^39^ hippocampus,^40,41^ amygdala and pallidum volumes.^42^ Given the interaction between these brain areas, motor and cognitive reserves (MCR) can rely on multiple, and even similar, brain domains interacting simultaneously to maintain function. Thus, a more appropriate term would be a combined term, MCR.

**Figure 1:**
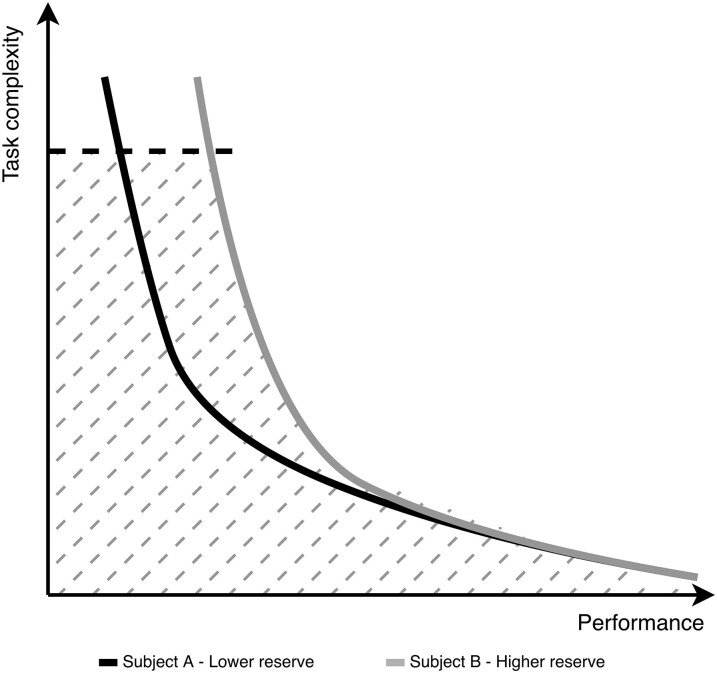
**Theoretical illustration of reserve capacity framework as derived from observing progression in completing challenging tasks.** The *y*-axis illustrates a scaled task complexity and the *x*-axis the performance (can also be viewed as how well a subject handled the task). The solid curves illustrate an expected completion of the task by two subjects: one with higher reserve capacity (grey) than the other (black). The horizontal dashed line represents a stop-point of the test. The AUC (between the axes, dashed line and solid curve) represents the reserve capacity we seek to measure. Current reserve proxies estimate one point on the subject's curve since the tests’ difficulty is fixed. In our approach, by manipulating the challenges during the test, the reserve can be seen as the AUC, i.e. individual progression in an increasing challenging test which induces demand on the MCR, we can quantify a more granulated proxy for the unobserved reserve capacity.

The use of various proxies to define motor or cognitive proxies is the consequence of the lack of alternatives for direct measurement, but this approach has limitations. It only reflects a given point in time, lacks description or capture of compensatory mechanisms and any dynamic properties of reserve.^43^ To circumvent this drawback, the residual approach was introduced in reserve research.^26^ This approach quantifies reserve as the variance in performance that remains unexplained after accounting for brain pathology or age-related structural changes. It represents the ‘residual’ beyond what would be predicted by measurable brain status.^44^ This method, however, assumes linearity and does not account for non-linear associations or collinearity,^38,45-48^ is sensitive to model specification and included covariates and maybe less accurate when compensatory mechanism is at play (such as dopamine transporter down-regulation).^49^ Thus, here, we set out to develop an adaptive quantitative test that can evaluate reserve by measuring the projected MCR under more severe decline, i.e. when more MCR allocation is needed (Fig. 1), potentially providing a predictive and prognostic measure. To achieve this, we used an approach similar to the one applied in other physiological reserve systems (cardiology, renal, etc.) by building a dedicated challenge test or ‘stress test’ that will explore the ability of a person to perform complex motor–cognitive tasks with increased graded difficulty that requires the utilization of increasing motor and cognitive resources. Here we report on the validation of this novel MCR index in adults with varied reserve capacities due to neurodegeneration and aging. Validation was explored in terms of: (i) model face validity, (ii) known-groups validity and (iii) construct validity against commonly used motor and cognitive proxies and pre-defined MRI-derived structural measures. We expected the MCR index to be sensitive to motor cognitive challenges and clinical status while correlating with the commonly used motor and cognitive proxies as well as specific brain regions’ volumes associated with motor or cognitive reserve proxies.

## Materials and methods

### Participants and data collection

To create and examine the MCR index, we recruited a group of participants with assumingly a broad spectrum of reserve capacity. This variance is reflected through the diverse sample, including young and older adults with an array of motor cognitive abilities as well as individuals diagnosed with neurodegeneration-related conditions [such as Parkinson’s disease, Alzheimer’s disease, dementia with Lewy bodies and mild cognitive impairment (MCI)]. Participants were enrolled if they were above 18 years of age, with no upper age limit and able to walk for 5 min without relying on any assistive device. Participants were included in the healthy group if they were not diagnosed with neurodegeneration or other medical condition as diagnosed by a movement disorders specialist or cognitive neurologist and did not consume medication to enhance cognition (i.e. methylphenidate or Selective Serotonin Reuptake Inhibitors and Serotonin Norepinephrine Reuptake Inhibitors). Participants with a wide range of diagnoses were included in the diagnosed group to enable building an MCR index sensitive to a broad spectrum of neurodegenerative processes. All patients were tested while on their regular medications in order to assess performance in their usual clinical state. Data from 70% of the participants were assigned to the training set (which resulted in 41 healthy and 57 diagnosed) and the remaining 30% to the validation set (27 healthy and 19 diagnosed). Another external validation cohort of *N* = 27 healthy subjects was recruited to perform both the MCR test and an MRI scan. This external validation set was used to validate the MCR index against MRI-based proxies. The study was approved by the local ethical committee and was performed according to the principles of the Declaration of Helsinki. All participants gave their informed written consent prior to participation, for patients with dementia who were not eligible to signing a consent, permission was provided by a legal guardian.

### MCR stress test set-up and protocol

The developed ‘stress test’ system includes a virtual reality (VR) simulation, a camera for motion capture (Intel RealSense), a treadmill and Inertial Measurement Units (IMUs) with three-dimensional (3D) accelerometers and gyroscopes (Opal by APDM Inc., Portland, OR, USA). The VR is used as the platform for providing the cognitive and motor tasks while the participant walks on the treadmill. This system is based on a training platform developed in Tel Aviv Sourasky Medical Center (TASMC),^50^ which was altered to expose subjects to a challenging motor–cognitive ‘stress test.’ The IMUs, placed on the participant's bilateral ankles, were used to collect the movement and performance gait measures while the camera provided information on the location and position of the participants’ feet and projected this information into the VR simulation to provide visual feedback. The first walk served as a familiarization trial for walking on the treadmill. During this 1-min walk, the participants’ preferred walking speed (m/s) was set to be used during the test as their baseline.

**Figure 2:**
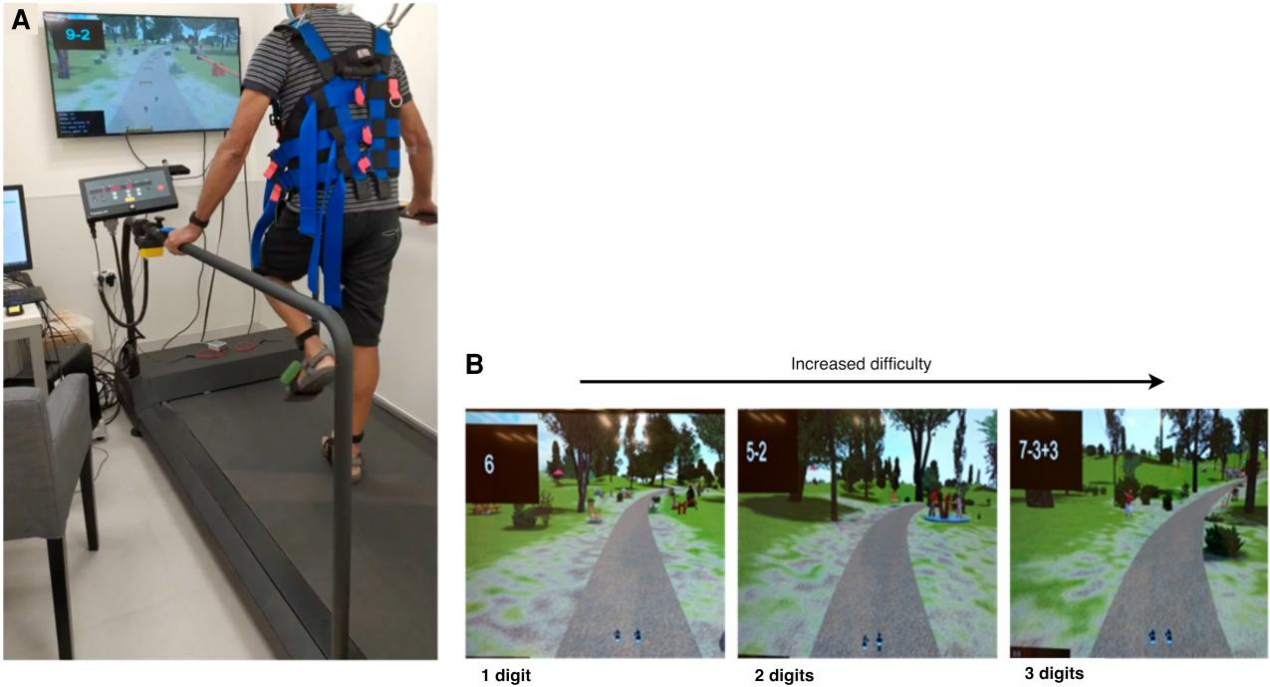
**Illustration of the MCR stress test platform. (A)** A photo of a live session while a subject completes the test (the subject consent was provided for the photo). The set-up is a treadmill with a screen placed in front of the subject. By placing wearable markers on the shoes, the participant can see a projection of their feet on the screen and their leg movement is translated to walking in the virtual trail. During a trial, motor, cognitive or simultaneous virtual challenges are projected. Motor challenges are virtual obstacles on the screen which the participant needs to pass. Cognitive challenges are arithmetic challenges located at the top-left corner of the screen. The participant is asked to press one of two buttons strapped to the handlebars of the treadmill to indicate if the expression sums to a value ≤4 or >4. **(B)** A zoom-in of the screen of three different levels of cognitive challenges.

The test is composed of a series of 16 walking trials ranging between 0.45 and 2 min for a total of ∼30 min for a complete test. Although all trials involve both domains, they are classified as motor-focused trials, cognitive-focused trials or combined motor–cognitive trials according to the challenges displayed in the VR simulation. In the *motor-focused* trials, the participant is asked to negotiate virtual obstacles while walking on the treadmill at different walking speeds relative to the determined preferred walking speed. In the *cognitive-focused* trials, the participant walks on the treadmill while arithmetic questions in various difficulty levels (e.g. number of digits and display times) are displayed. Using a response button, the participant is asked to press the button strapped to the treadmill's handlebars to indicate if the expression sums to a value ≤4 or >4 (Fig. 2A and B), and the answer is recorded. This simple cognitive response selection task was chosen to minimize bias of education and IQ. The combined *motor–cognitive* trials incorporated virtual obstacles navigation while performing the cognitive tasks (Appendices A and B). The first three trials of the test were fixed in order for all subjects to have the same learning experience (motor-focused, cognitive-focused, followed by a motor–cognitive trial). In the other 13 trials, the type of challenges was randomized in order.

The measurements collected during the MCR stress test include performance measures such as success rate in passing cognitive or motor challenges, reaction time in the cognitive tasks and gait measurements obtained from the wearable sensors, including features such as mean and standard deviation (SD) of step length, Gyro amplitude at mid-swing and several symmetry features.^51^ Additional details on the MCR stress test protocol and features collection are provided in Appendices A and B.

### MCR index computation

The MCR index was calculated using the previously developed stress test performance scoring framework,^52^ which is a semi-supervised machine learning tool that models expected performance in a stress test set-up. During the computation of the MCR index, several outputs were obtained and used for index validation purposes. Those include *standardized performance scores* for each subject in each challenge level. From these scores, a 3D ‘performance plane’ was created for each subject as a function of the two dimensions of difficulty levels—the cognitive and motor domains. A *reference performance plane* was also created using the *median scores* of the healthy control group from the training set.^52^ Similarly, we constructed a performance plane for the diagnosed patients group. The reference plane sets the expected performance for a new subject. Similarity to the reference plane represents intact reserve, while a disparity indicates MCR reserve deficit.

The MCR index was then calculated as the average difference between an individual's performance plane and the reference plane. The MCR index is scaled to range between 0 and 100, with higher values reflecting greater reserve (estimated minimum and maximum values were retained to scale the test set's scores). For more detailed information on the MCR index computation see Supplementary Appendix C and Supplementary Figs S1 and S2.

### Clinical proxies of motor and cognitive reserve

We examined several commonly used motor and cognitive tests that have previously been reported in reserve studies.^18-23^ Years of education was included as a proxy of reserve to evaluate construct validity. We also evaluated age and performance on neuropsychological tests such as the Montreal Cognitive Assessment (MoCA)^53^ which assess global cognitive function, The Trail Making Test (TMT) parts A and B^54^ and the Stroop interference,^55^ which evaluate executive function and response inhibition, to assess association with the MCR. Preferred walking speed was used as a proxy of motor capacity and frailty.^56^ All metrics were transformed such that higher values indicate better results.

### MRI data acquisition

Magnetic resonance (MR) data were acquired using a 3-T Magnetom Prisma® (Siemens, Erlangen, Germany) MR scanner equipped with a 20 channels phased-array head coil. The MRI protocol included: (i) a high-resolution 3D magnetization-prepared two rapid gradient echo (MP2RAGE) sequence (TR/TE/TI_1_/TI_2_ = 5000/3.43/803/2500 ms; FA_1_/FA_2_ = 4/5°, voxel size = 1 × 1 × 1 mm^3^, acquisition time: 7:07 min), (ii) axial T2-fluid attenuated inversion recovery (FLAIR) (TR/TE/TI = 8000/117/2370 ms; FA = 150°; voxel size = 0.8 × 0.8 × 5 mm^3^, acquisition time: 2:58 min).

### MRI data processing

Assessment of T2-FLAIR images of all participants by an experienced neuro-radiologist revealed no significant incidental findings that would lead to excluding participants from further analysis. Volumetric data processing was performed on the derived T1-weighted images acquired as previously described.^57^ Briefly, the computational analysis toolbox (CAT12, http://www.neuro.uni-jena.de/cat/), an extension for statistical parametric mapping (SPM12, https://www.fil.ion.ucl.ac.uk/spm/software/spm12/) was applied to the acquired T1-weighted images of all participants. This yielded whole-brain grey matter (GM), white matter (WM) and CSF probability maps,^57^ as well as deep grey matter structure volumes based on a multi-atlas approach to produce 10 segments: left and right caudate, putamen, hippocampus, pallidum and amygdala. The obtained MRI brain volumetric metrics were adjusted to the measured total intracranial volume of each subject individually.

### Statistical analysis

The statistical analysis focused on three key validation components: (i) model's face validity—to evaluate if the test measures the addressed concept, (ii) known-groups validity—ability to discriminate high and low reserve and (iii) construct validity—the test correlates with the current used proxies. Each component assessed a distinct aspect of the index's performance using behavioural, clinical and imaging data, as detailed in the subsections below.

1. Model's face validity—The subjects’ performance planes were modelled with a linear regression where performance scores (dependent variable) are a function of two ordinal explanatory variables—the cognitive and motor challenges—and their interaction with the group variable. We expected a deterioration in scores with an increased challenge in both groups yet that healthy subjects will have only minor differences between difficulty levels. Thus, the regression model's coefficients are reported with their corresponding *t*-test *P*-values.
2. Known-groups validity—This was performed by reviewing each group's performance scores’ distribution and their rate of overlap.^58,59^ Specifically, for each combination of cognitive and motor difficulty levels, we calculated the area of overlap between the density functions of performance scores for the diagnosed and healthy control groups. Further, using the test set, the accuracy of the MCR index in identifying the subjects’ current neurological status was explored by fitting a univariate logistic model and calculating the area under the curve (AUC) alongside 95% De-long confidence intervals (CIs).^60,61^
3. Construct validity—Spearman's *ρ* correlations were used to assess associations of the MCR index against the following proxies: years of formal education and preferred walking speed on a treadmill. We also assessed association with age, TMT parts A and B, Stroop interference and MoCA. In addition, the associations of the MCR index with MRI-derived volumetric brain metrics previously associated with reserve^36-42^ (i.e. whole-brain GM and WM, as well as volumes of the hippocampus, pallidum, putamen, caudate, amygdala and superior-and-inferior frontal regions) were investigated. This exploration was done with an external validation cohort of 27 subjects who completed the MCR stress test and an MRI scan within a three-month window. *P*-values were adjusted to control for a false discovery rate at a level of 0.05 with the Benjamini–Hochberg (BH) procedure.^62^

## Results

A total of *144* participants (age range of 18–85, 50% women) were recruited from the Neurological Institute at the TASMC. Participants’ characteristics are detailed in Table 1.

**Table 1:** Participant characteristics

		Healthy controls (*N* = 68)	Neurological patients (*N* = 76)	*P*-value (Wilcoxon or *χ*^2^ test)
Sex	Female	36 (52.9%)	37 (48.7%)	0.731
Age at enrolment	Mean (SD)	51.1 (17.7)	66.2 (10.6)	<0.001
	Median [min, max]	52.5 [17.6, 82.3]	68.2 [31.7, 85.0]	
Years of formal education	Mean (SD)	16.4 (2.54)	15.6 (3.60)	0.072
	Median [min, max]	16.0 [11.0, 22.0]	16.0 [10.0, 25.0]	
Disease duration (years)	Mean (SD)	0 (0)	4.09 (5.55)	Not applicable
	Median [min, max]	0 [0, 0]	2.00 [0, 30.0]	
Stroop word (s)	Mean (SD)	94.8 (17.0)	82.8 (15.6)	<0.001
	Median [min, max]	96.0 [52.0, 124]	84.0 [50.0, 113]	
Stroop colour (s)	Mean (SD)	95.0 (18.4)	83.6 (16.0)	<0.001
	Median [min, max]	96.0 [44.0, 130]	85.0 [45.0, 112]	
Stroop interference (s)	Mean (SD)	44.7 (13.8)	33.7 (14.4)	<0.001
	Median [min, max]	44.0 [15.0, 88.0]	32.0 [10.0, 98.0]	
TMT part A (s)	Mean (SD)	42.3 (18.3)	77.6 (44.7)	<0.001
	Median [min, max]	38.6 [13.8, 99.8]	64.8 [21.0, 235]	
TMT part B (s)	Mean (SD)	80.2 (32.8)	121 (52.6)	<0.001
	Median [min, max]	71.9 [33.0, 195]	112 [48.5, 307]	
TMT B/A (% change)	Mean (SD)	200 (55.1)	180 (55.3)	0.057
	Median [min, max]	193 [75.1, 355]	181 [57.4, 357]	
MoCA	Mean (SD)	27.5 (2.69)	25.3 (2.69)	<0.001
	Median [min, max]	28.0 [18.0, 30.0]	26.0 [19.0, 29.0]	
Preferred walking speed on treadmill (km/h)	Mean (SD)	2.93 (0.300)	2.41 (0.491)	<0.001
	Median [min, max]	3.00 [2.00, 3.80]	2.45 [1.20, 3.40]	
MCR index	Mean (SD)	74.9 (13.5)	45.5 (14.9)	<0.001
	Median [min, max]	76.7 [45.0, 100]	46.8 [0, 82.8]	

Continuous variables are presented with mean (SD) and median [min, max], and were compared between the control and diagnosed groups using the Wilcoxon test. Categorical variables are presented as counts (percentages) and were compared using the chi-square test. *P*-values are provided for all tests. TMT = Trail making test; MoCA = Montreal cognitive assessment, Preferred walking speed was evaluated on the treadmill reflected as Km/h.

### Model's face validity

**Figure 3:**
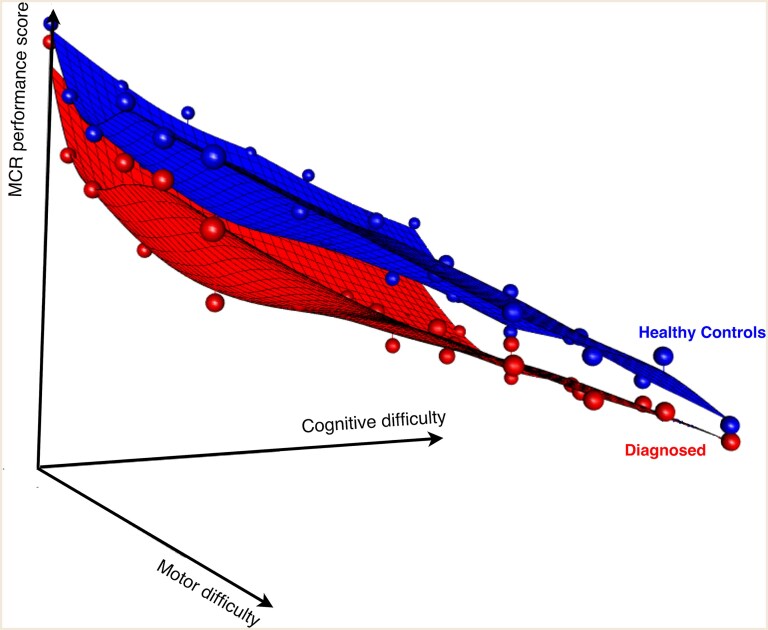
**Performance planes per healthy control (top) and diagnosed (bottom) group.** The vertical axis is the MCR performance score as a function of both motor and cognitive difficulty loads (horizontal axes). A higher performance score reflects a better result. The reference performance plane summarizes the expected performance in the MCR stress test, as observed among healthy control (in blue) participants in the training set. Since task difficulty increases along both cognitive and motor dimensions, this reference takes the form of a 3D plane across those domains. Hereafter, a test subject's MCR score will be computed as the average distance between their observed performance scores and the expected scores defined by this reference plane. A smaller distance indicates performance aligned with healthy norms, while larger distances reflect potential deficits in MCR. See Supplementary Table S2 for the difficulty load hierarchy of the motor and cognitive domains. See Fig. 4 for the performance scores’ distribution per group in each of the difficulty levels.

Figure 3 presents the performance planes for the healthy control and diagnosed patient groups. The modelling of the performance planes revealed statistically significant main effects but no statistically significant interactions. Specifically, the group effect was 0.589 (*P* < 0.001), indicating that the expected score on each challenge was higher by 0.589, on average, in the healthy group. The motor difficulty domain had an effect of −0.303 (*P* < 0.001), while the cognitive difficulty domain had an effect of −0.262 (*P* < 0.001), indicating that an increase in difficulty level resulted in a decline of −0.303 and −0.262 on average, respectively.

### Known-groups validity

**Figure 4:**
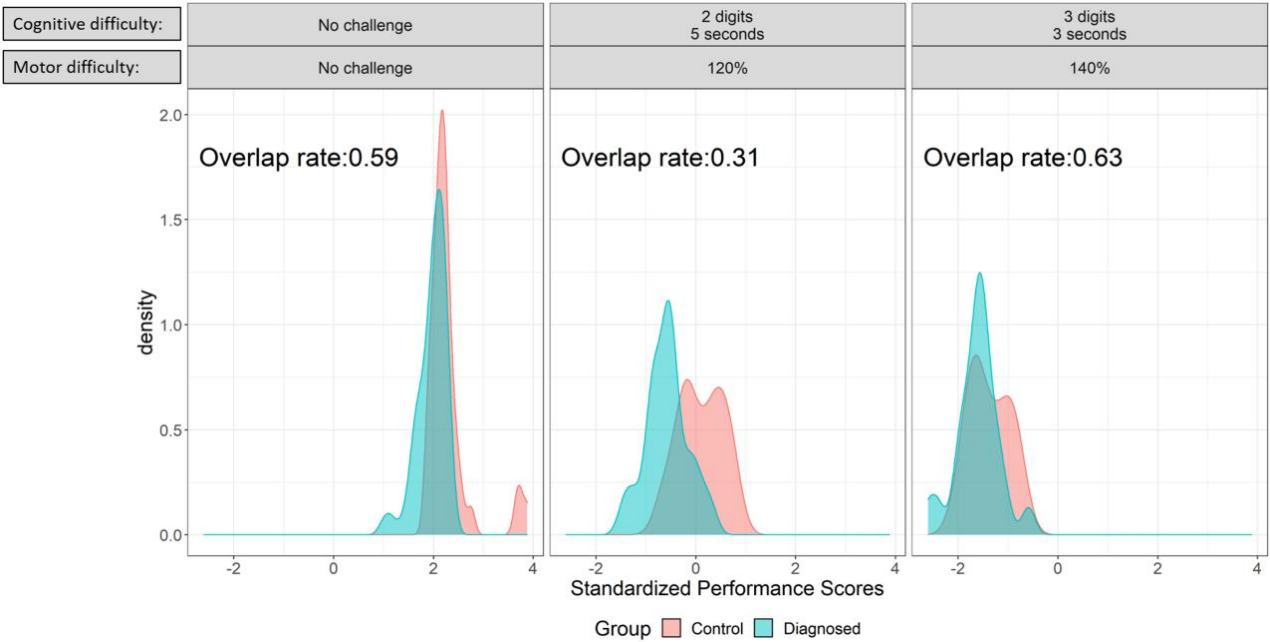
**Standardized performance scores (*Z*-scores) distribution and overlap rates for selected challenges levels.** The distribution of performance scores for selected challenges in the healthy control group (blue) and the neurological patient group (red). The cognitive domain challenge levels are a combination of number of digits and display time of the challenge. The motor domain challenge levels are the relative speed to the preferred walking speed in percentage. Overlap rates of the standardized performance scores’ (*Z*-scores) densities between the neurological patients and the healthy control groups (i.e. proportion of overlapping area between the density distributions) are noted next to the densities. Overlap rates are higher in the easiest challenge and on the most challenging level, but decrease in the moderate challenge level, indicating lower similarity in performance scores between the two groups. The ‘No challenge’ refers to a time segment in which the subject only walked on the treadmill. A higher performance score reflects a better result. See Supplementary Appendix A and Table S1 for further information on the MCR stress test protocol and difficulty levels induced.

In Fig. 4, we present the density of performance scores for each group based on challenge levels: no challenge, the most difficult level (treadmill speed of 140% relative to the preferred walking speed and arithmetic challenges of three digits displayed for 3 s) and a moderate challenge level consisting of speed of 120% and two digits displayed for 5 s. The figure displays the overlap rate at each difficulty level illustrating a U-shaped overlap, with both groups scoring similarly at the easy difficulty level (high overlap of 0.59). As the challenge increased to a moderate level, the groups started to differ in performance, with the healthy control group scoring better (lower overlap rate of 0.31). However, as difficulty increases, the performance of subjects’ in both groups deteriorated (overlap rate of 0.63). Additional examples are shown in Supplementary Appendix D, Fig. S3.

Known-groups validity analysis also showed that the MCR index had an AUC level of 0.89 (95% CI: 0.7936–0.992) in classifying healthy subjects from neurological patients, which was significantly higher than all other common proxies (Table 2). ROC curves of all the MCR measurements are provided in Supplementary  Appendix E, Fig. S4.

**Table 2:** AUC levels with their corresponding 95% CI for various MCR proxies

Measurement	AUC level [95% De-Long CI]	*P*-value (one-sided De-Long AUC test)
MCR index	0.893 [0.794–0.992]	Not applicable
Age at enrolment (*y*)	0.713 [0.561–0.865]	<0.001
Years of formal education	0.684 [0.468–0.899]	<0.001
MoCA	0.780 [0.646–0.915]	<0.001
TMT part A (s)	0.808 [0.673–0.943]	0.009
TMT part B (s)	0.825 [0.694–0.955]	0.006
Stroop word	0.714 [0.541–0.888]	0.002
Stroop colour	0.710 [0.539–0.881]	<0.001
Stroop word-colour	0.755 [0.595–0.916]	<0.001
Preferred walking speed on a treadmill	0.755 [0.611–0.899]	<0.001

*P*-value derived from applying a one-sided De-Long AUC test comparing each of the MCR proxies to the MCR index.

### Construct validity

**Figure 5:**
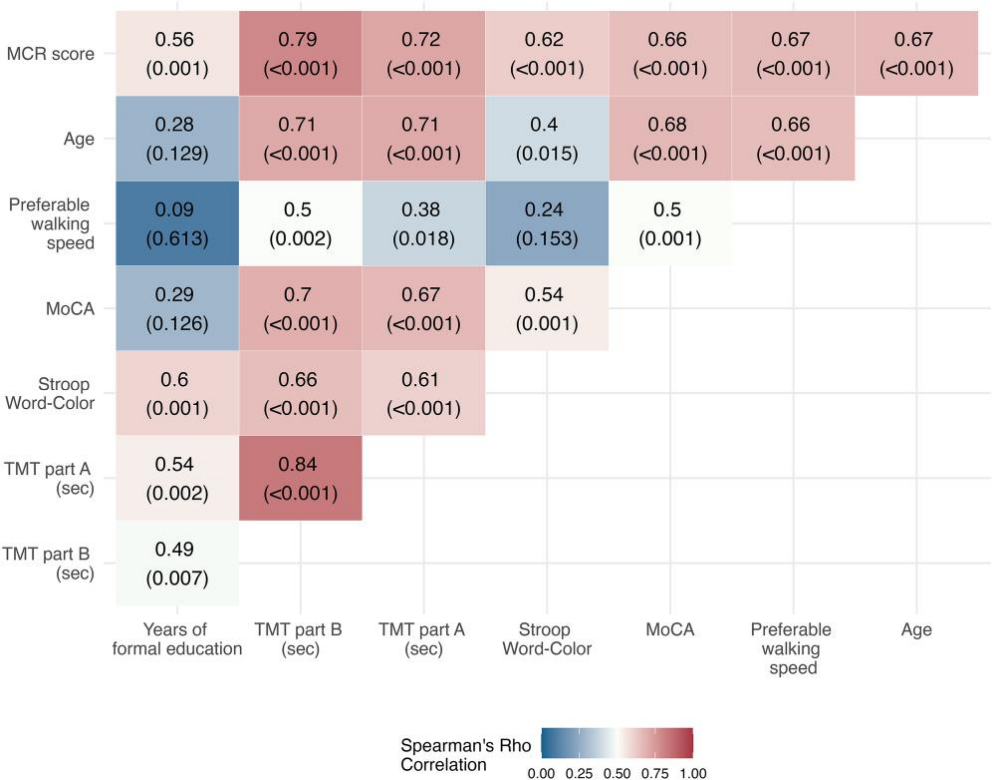
**Heat map of correlations between the MCR index and clinical measures.** Spearman's *ρ* correlation coefficient (*P*-value) is presented for each pair of proxies. The colour indicates the correlation value. All proxies were transformed such that high values represent worse state (*N* = 46).

Figure 5 presents the examined correlations. The MCR score shows high and statistically significant correlations with the age and the most studied proxy years of formal education (0.67; adjusted *P* < 0.001 and 0.55; adjusted *P* < 0.001, respectively). Correlations with additional metrics ranged between 0.56 and 0.79 and were statistically significant after multiplicity corrections (all adjusted *P* < 0.05).

**Figure 6:**
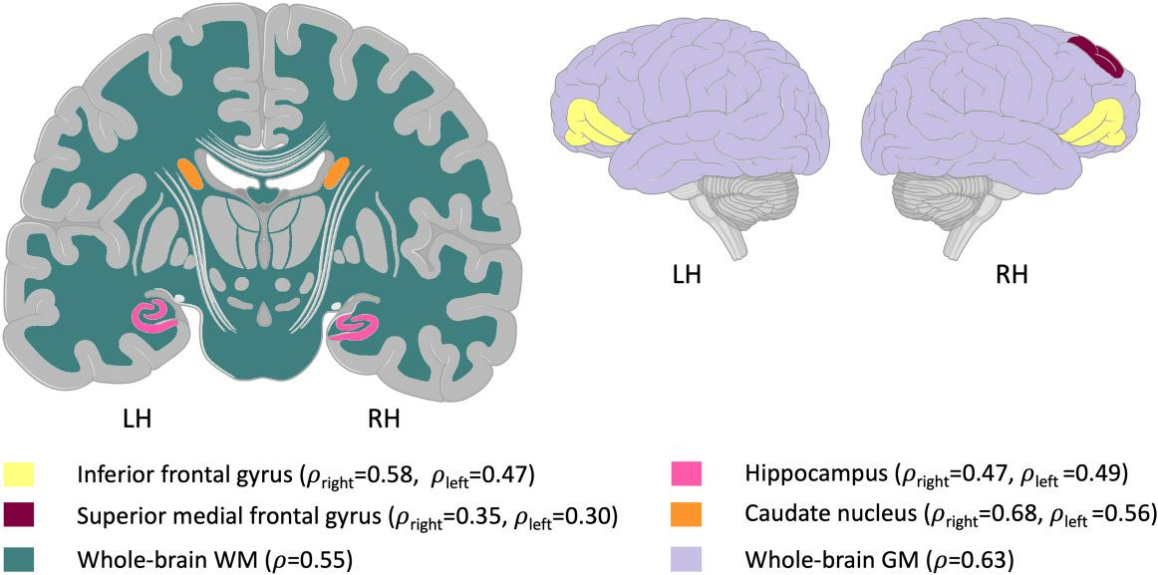
**Brain regions with statistically significant correlation to the MCR index.** Regions that yielded a statistically significant BH adjusted *P*-value (adjusted *P* < 0.05) for their Spearman's *ρ* correlation coefficient (*ρ*) with the MCR index are highlighted. Left amygdala not depicted on the shown brain slices (*N* = 27). LH = Left-hemispheric; RH = Right-hemispheric.

We used MRI data from 27 healthy subjects (mean age: 60.3 ± 10.8 years, range: 45–80 years) who completed both the MCR test and an MRI scan (mean interval between MCR and MRI: 9.5 weeks). Figure 6 highlights the brain regions that were significantly correlated with the MCR index (*P* < 0.05) after multiplicity adjustment. These areas include the caudate nucleus (adjusted *P* = 0.018 and 0.004 for left and right sides, respectively), the right bilateral superior medial frontal gyrus (adjusted *P* = 0.041), the inferior-frontal gyrus (adjusted *P* = 0.043 and 0.017), hippocampus (adjusted *P* = 0.041 and 0.043), the left amygdala (adjusted *P* = 0.043), whole-brain grey matter (adjusted *P* = 0.009) and whole-brain WM (adjusted *P* = 0.018) volumes. The correlation results of these and other tested measures are detailed in Supplementary  Appendix F, Table S3, which includes *P*-values before and after multiplicity adjustment. In Supplementary  Appendix G, Table S4, we present a *post hoc* analysis that explores that the correlations with the MRI data could have not been solely explained by age.

## Discussion

In this study, we tested the ability of a combined motor–cognitive challenge test to quantitatively assess the theoretical MCR capacity. Our analysis of the MCR index aimed to evaluate and verify that this index captures aspects of MCR capacity. Our findings show: (i) that the MCR test has face validity reflected by the decline in performance with greater difficulty levels and in the face of clinically diagnosed disease (assumed lower reserve); (ii) the MCR index was able to correctly identify individuals with neurodegeneration with higher sensitivity than the current used proxies; (iii) the index correlated with well-established clinical and behavioural reserve proxies in both the motor and cognitive domains and with brain volumetric measures associated with reserve.

In line with our assumptions, performance scores generated at each difficulty level of the test worsened with increased challenge (as shown by the statistically significant negative slope of the planes shown in Fig. 3). Moreover, as hypothesized, we found that the median score in each difficulty level for the patients’ group was worse than that of the healthy control group. In the performance scores per difficulty level's densities, we noticed some overlap between the two groups, which was higher in the lower difficulty levels (demonstrated with a high overlap rate). However, even when no challenge was induced, the overlap was not complete due to variance known to be present among subjects with neurodegeneration.^63^ Based on previous evidence,^28,56,64^ our hypothesis was that as both motor and cognitive difficulty loads increase, even healthy subjects would need to recruit additional resources to deal with the challenges. We posited that all individuals can reach a tipping point when the challenge is sufficient, at which point no additional resource allocation is feasible, and performance will decline. Our findings show that the overlap rate decreased as the difficulty increased up-until mid-level challenges. At this time, individuals with high reserve performed well, and those with low reserve already deteriorated. As the difficulty further increased, all subjects’ performance deteriorated, indicating that the test was sufficiently challenging even for healthy subjects, so that they were required to recruit additional resources. The overlap in the highest difficulty level suggests that even some healthy participants reached their ‘tipping point,’ and the point of failure differs according to capacity (see Fig. 4). This interpretation aligns with the theoretical threshold model of reserve, which defines it as a finite capacity that can be drawn upon to maintain function despite accumulating pathology.^65^ Based on this theoretical concept, symptoms become visible when reserve is diminished or exhausted. Thus, as hypothesized, reserve, as measured with MCR, was lower in the diagnosed from non-diagnosed. Our analysis showed that in comparison to the commonly used proxies of cognitive and motor reserves, the MCR was significantly better in classifying healthy controls from diagnosed patients. The combined motor and cognitive tests were more sensitive than each of the focused tests (recall Table 2 and Supplementary Fig. S4). However, their true utility would be in identifying individuals with low reserve through failed compensatory performance that have imminent disease. Identifying early markers of neurodegeneration before diagnosis could open the opportunity for prevention treatments. This should be explored in future studies.

Various proxies have been suggested to reflect a subject's MCR capacities. Among these, age, although not a direct proxy, has a complex relationship with MCR. Reserve may be diminished due to age but at the same token, reserve varies enormously between individuals of the same age while some younger people may have low reserve due to limited educational or social opportunities and some older adults can maintain high reserve through continued learning and engagement.^66^ In this study, both groups included younger and older adults with similar age distribution although a difference in mean age. While age differences between our examined groups may introduce confounding, in this study, age serves as a reference point rather than a covariate to adjust for. Importantly, our known-groups validity analysis indicates that age alone does not fully account for the observed group differences. In addition, a strong correlation was found between age and the MCR, but not a perfect one suggesting that the index captures additional variance, probably stemming from other relevant clinical sources beyond age alone.

In line with this, Nucci *et al*.^67^ provided a review of cognitive reserve proxies, which could be broadly categorized into educational, occupational, leisure activity, intelligence-based proxies, or a combination of the four. The limitation of those proxies, including the Cognitive Reserve Index questionnaire suggested by Nucci *et al*.,^67^ is that they capture a static state of a subject at one point in time and ignore compensatory mechanisms, which cannot be accounted for with a single-level test. To verify that our proposed novel index quantifies MCR, we explored its correlation with common behavioural proxies in both cognitive and motor domains. The MCR index was significantly associated with all of the commonly used proxies, such as years of education, and preferred walking speed. The moderate association, however, also suggests that the relation is not complete and that MCR likely measures additional aspects that the individual test may not. Moreover, the MCR index correlated with all proxies regardless of the domain (motor or cognitive) and was also associated with motor and cognitive functional brain areas. Although age and the MCR index are strongly correlated (*r* = 0.67), their significant brain associations do not fully overlap (Supplementary Appendix G). When both measures were adjusted for age, only the left and right caudate nuclei remained significant, while several other regions showed reduced but still noticeable effects, likely underpowered due to the small healthy control hold-out sample, indicating that the MCR index captures variance beyond age alone. These associations support the idea that the suggested index captures neuronal (central, peripheral and autonomic nervous systems) and musculoskeletal functions in both domains and has the ability to unmask compensation by either domain.

In this study, we examined correlations between brain structural measures that are widely associated with cognitive and motor performance in various neurological conditions (e.g. Alzheimer’s disease, Parkinson’s disease and MCI), and the MCR index. This analysis was conducted on a hold-out dataset to evaluate the construct validity of the proposed index. We found statistically significant correlations with areas known to be involved in memory and executive function, particularly those involved in planning, learning and decision-making.^37,42,68^ The caudate nucleus, hippocampus, amygdala and frontal regions were previously shown to correlate with IQ, a well-studied clinical proxy of cognitive reserve.^38^ The index also correlated with total GM and WM, which are reflective of general brain reserve.^69^ A weak correlation was also found with the amygdala, which is involved with emotional aspects and coping with difficult situations and was previously referred to in the context of psychologic resiliance.^70^ The MCR, with its gamification properties, offers another level of engagement which relies on motivation and competitiveness. The correlation between the MCR and the amygdala may suggest to tap into these aspects. Such resilience differs from cognitive or motor reserve, but similarly depletes with aging and neurodegeneration.^71^ It would be interesting to investigate further whether these correlations would be stronger among participants diagnosed with neurological conditions who may experience the test as more complex or provoking anxiety, as another aspect of resilience. Indeed, these correlations may reflect a neural network involvment. The MCR may be sensitive to large -scale higher brain processes that can be achieved from advanced whole-brain-network analyses in future studies.

This study has several limitations. First, our limited sample size restricted the inference in terms of statistical power obtained. In addition, as the MCR test is essentially a performance-based test, it relies on participants’ participation, motivation, engagement and fatigue levels. These aspects were not evaluated in this proof-of-concept study. In future work, this can be assessed by using structured questionnaires on engagement or by taking into account the time of completing the test, similar to the Bruce protocol of the ECG treadmill stress test.^72^ Future work should also investigate whether a higher-resolution classification can identify when a subject is closer to the point of deterioration and the trajectory of the performance plane in each of the domains, in order to tailor treatment specifically to the subject. In addition, enhancing the MCR index model by incorporating additional or alternative features—such as reaction time variability or additional cognitive tasks—may add sensitivity and robustness.^73,74^ Training the model on a broader and more representative general population is also a key step forward to improve generalizability and reduce dependency on proxies such as education or IQ. In recent years, there is a shift in the field of neurodegeneration as a result of the identification of biological markers of disease.^75-79^ These important developments will allow for the provision of preventive or disease modifying interventions to those at risk. However, currently, tests for the biological markers of alpha-synuclein, Tau and amyloid are not widely available, are invasive and expensive, limiting access and scalable screening potential. MCR has a potential to become a sensitive quantitative functional test that can be scalable, low-cost and low burden on both the testee and the tester to eventually improve our ability to identify individual's risk for developing neurodegenerative conditions or functional deterioration as well as providing prognostic information in a personalized approach. This proof-of-concept study represents a first step toward that goal. Longitudinal research is needed to examine whether the MCR index value changes with neurological deterioration and to assess its predictive abilities and sensitivity. It would be interesting to determine if the index can be specific to cognitive or motor decline or incident of dementia, as well as whether it differs between sub-populations of neurodegeneration such as Parkinson’s disease and Alzheimer’s disease, which our limited sample size did not allow us to examine. Nonetheless, the presented work provides, for the first time, a novel quantifiable assessment of motor and cognitive performance while providing a normative reference on an individual level reflecting quantitation of capacity. Such assessment provides a more anchored window into reserve and has the potential to alert on possible risk.

## Conclusions

The study explored a novel method to assess MCR. The test combines cognitive and motor challenges to measure MCR in a graded difficulty ‘stress test’. The model found correlations between the MCR index and well-established clinical, behavioural and imaging proxies of reserve. The MCR index depicts MCR capacities by quantifying performance during a challenging multi-level test and, therefore, may also allude to compensatory mechanisms. Similar to the way stress tests for renal and pulmonary reserve are used to screen subjects with an underlined dysfunction, perhaps, the MCR stress test can be used to identify individuals with a lower reserve capacity, who are at greater risk for neurodegenerative related conditions.

## Supplementary Material

fcaf412_Supplementary_Data

## Data Availability

The data supporting the findings reported here will be available upon reasonable request by researchers who meet the criteria for access to confidential data. The code used to implement the MCR index model is based on the algorithm published in Kozlovski *et al*. and is publicly available at: https://github.com/TalKozlovski/STEPS.
