# Supplementary material for: Quantifying motor–cognitive reserve using a novel multi-modal stress test
Source: Brain Commun. 2025 Oct 24;7(6):fcaf412. doi: 10.1093/braincomms/fcaf412 (PMC12613160; doi:10.1093/braincomms/fcaf412)
Supplement: fcaf412_Supplementary_Data [file fcaf412_supplementary_data.docx]

**Appendix A -MCR stress test protocol**

Let define an "experiment unit" as a walking sequence that shares the same properties of the following three features: *Speed* $\in\{0, 1, 0.8, 1.2, 1.3, 1.4\}$ relative to the preferable walking speed (pWS) of each subject (*Speed* defines a motor domain's difficulty level), *Digits* $\in\{0, 1, 2, 3\}$, and *Duration* of {$3, 5\}$ seconds for the question to appear on the screen (the two latter defines together a cognitive domain's difficulty level). When motor or cognitive difficulty levels are set to "0" it represents that only cognitive or motor challenges appeared on the screen, respectively. If both are set to "0", then it stands for a walking sequence from the beginning of a trial along 18 meters until the subject reaches the first challenge on screen. We refer to it as usual walk.

*Motor-focused* trials are consist from a 18 meters walk in pWS, then the treadmill speed is set to 80% of the pWS, and after six hurdles the speed increases, first to 100%, then to 110%, 120%, 130% & 140% relative to the subject pWS, respectively. *Cognitive-focused* trials display 4 sections with 6 arithmetic question each, involves difficulty combinations of 2 or 3 digits and 3 or 5 seconds of display.

*Motor-cognitive* trials combine hurdles and arithmetic challenges, where the speed is fixed along the trial (one of $\{0, 1, 0.8, 1.2, 1.3, 1.4\}$), and two sections with arithmetic questions are displayed, in both the number of digits is the same (1, 2, or 3 digits), and only the display duration is changing.

The first three trials are fixed in order for all subjects to have the same learning experience (motor, cognitive, following with a motor-cognitive trial) and expose the subject to the easiest difficulty loads. The other 13 trials were randomized, and each consist of a motor trial, a cognitive trial, and motor-cognitive trials in different challenge properties.

The MCR test was designed to assess multiple motor and cognitive domains even in the motor-focused and the cognitive-focused trials. The simple arithmetic task primarily engages working memory, processing speed, and attention. Participants also used simple memory for task instructions. The obstacle negotiation task, though motor-focused, requires cognitive processing in executive function, attention, visuospatial processing, and working memory. Cognitive experts aimed to tap into multiple domains simultaneously to define reserve rather than focusing on a specific domain.

Additionally, the motor domain involves motor planning, coordination (such as foot height and step before and after the obstacle), and balance control during negotiation.

**Appendix B - Preprocessing**

Measures of gait and success rate are summarized with their average and SD value per experiment unit (definition in Supplementary A). An experiment unit can be replicated during a subject session in more than one walking trial. SD values of repeated measures were smaller for each subject than the overall of her assigned group; therefore, to ease calculations, repeated measures of the same difficulty level per subject were averaged. Thus, the repeated measures term in the STEPS framework is redundant. Motor and cognitive success measures were weighted for averaging according to the number of motor or cognitive challenges encountered on each walking sequence. Gait measures were weighted for averaging according to the total number of steps executed on each walking sequence.

Since base speed is a marker for neurodegeneration^1^, and we used this information to rank subject according to their medical status (more details in Supplementary C), we want to clean out its effect from the subjects' feature vectors to avoid our model to learn this dependency. Since we aim to model performance related to unobserved reserve deficit, i.e., to overcome compensation mechanisms that override symptoms presence, we do not want our model mainly to rely on base speed information. Therefore, gait measures (Mean Swing (Sec), Mean Stance (sec), Mean Double Support (sec), and Mean Step Length (sec)) were adjusted to base speed. The effect of base speed is cleaned using polynomial regression fitting^2^ (span of 0.75 and polynomial degree of 2). Missing values due to systematic absence were filled with the value 9 or -9 (Supplementary Table S1). The imputation does interfere with the algorithm fitting model since it is tree-based. This step was necessary to avoid missing values in the data input table. Last but not least, features that were found to be highly correlated with others were removed (randomly chosen from such correlated pair), i.e., if Spearman correlation's coefficient >0.95.

Table S1: List of features engineered

| **Variable** | **Description** | **Included in training of the model** | **Value imputed if missing** |
| --- | --- | --- | --- |
| Preferable walking speed (pWS) | Initialization parameter | FALSE | NA |
| Motor speed | Defines experiment unit of motor challenge level | FALSE | NA |
| cognitive number of digits | Defines experiment unit of cognitive challenge level | FALSE | NA |
| cognitive display duration | Defines experiment unit of cognitive challenge level | FALSE | NA |
| logit_MotorSuccess_Left | logit of percentage of hurdles successfully passes on the left side | TRUE | 9 |
| logit_MotorSuccess_Right | logit of percentage of hurdles successfully passes on the right side | TRUE | 9 |
| logit_MotorFails_Left | logit of percentage of hurdles failed passes on the left side | FALSE | 9 |
| logit_MotorFails_Right | logit of percentage of hurdles failed passes on the right side | FALSE | 9 |
| logit_MotorSkips_Left | logit of percentage of hurdles not attempted to pass on the left side | TRUE | 9 |
| logit_MotorSkips_Right | logit of percentage of hurdles not attempted to pass on the right side | TRUE | 9 |
| Mean_reactionTime | Average time to first press on the button from the question display | TRUE | -9 |
| Total_presses | Average of time at which one of the buttons were pressed during cognitive challenges at the same experiment unit | TRUE | -9 |
| logit_CogSuccess | logit of percentage of cognitive questions successfully solved | TRUE | 9 |
| logit_CogFails | logit of percentage of cognitive questions not successfully solved | FALSE | 9 |
| logit_CogSkips | logit of percentage of cognitive questions not attempted (without any button press) | TRUE | 9 |
| Mean_Swing_Time | Average time length of swing phase | TRUE | NA |
| Mean_Stance_Time | Average time length of stance phase | TRUE | NA |
| Mean_DoubleSupport_Time | Average time length of double support phase | TRUE | NA |
| Mean_MS_AMP | Average amplitude during mid-stance phase | TRUE | NA |
| SD_Swing_Time | SD of time length of swing phase | TRUE | NA |
| SD_Stance_Time | SD of time length of stance phase | TRUE | NA |
| SD_DoubleSupport_Time | SD of time length of double support phase | TRUE | NA |
| SD_MS_AMP | SD of amplitude during mid-stance phase | TRUE | NA |
| Mean_Step_Length | Average time length of a whole step | TRUE | NA |
| Mean phase of the stride (Mean_PoS) | 360 * (Mean_Step_Length/Stride_Length) | TRUE | NA |
| SD_PoS | SD of Mean_PoS | TRUE | NA |
| SD Absoulute value of PoS (ABS_PoS) | \|Mean_PoS - 180\| | TRUE | NA |
| Coefficient of variation of the phase [%] (Phi_CV) | SD_PoS / Mean_ PoS | TRUE | NA |
| Gait Asymmetry (Mean_GA) | 100 * \|ln(Swing_right_leg / Swing_left_leg)\| | TRUE | NA |
| Phase Coordination Index (PCI) | (100 *(mean_ABS_PoS) / (180)) + PoS_CV | TRUE | NA |

**Appendix C - MCR score computation with the STEPS framework**

To apply the STEPS framework, two sets are constructed: 1) Set $B$, the between-subjects set, that holds pairs of ordered subjects. That is $<x_{i},x_{j}>$ where subject $i$ is considered in a severe state in terms of MCR comparing to subject $j$. Each pair is extended to another set that holds all common difficulty levels between the two subjects, and 2) set $W$ that holds within-subject pairs of difficulty levels encountered by a subject during the MCR stress test.

The construction of the between-subjects set, $B$, was based on age, years of education (YE), and extensive clinical examinations the subjects went through in our lab on the same day they faced the MCR stress test. Subject $i$ is ranked higher (as in worsen state) than subject $j$ if $age_{i}\geq age_{j}$, and/or $YE_{i}\leq YE_{j}$ and if subject $i$ achieved lower scores (score difference is greater than the clinical minimal difference value^3^, or one SD unit) in at least 4 out 5 cognitive measures, and/or at least 2 out of 3 motor measures. The ranking was done separately for cognitive and motor domains. For the cognitive ranking we used measures of MoCA^4^, Stroop Test^5^, Trail Making Test^6^, and MMSE^7^. For the motor ranking, we used the score of UPDRS part III^8^, pWS, and information on falls in the past 6 months. For the full ranking mechanism of motor and cognitive domains, please see Appendix Figure S1, S2. Table S2 presents the complete difficulty levels and their hierarchy.

Other parameters to define are the weight parameters, $\lambda_{W}$ and $\lambda_{R}$, which according to the simulation presented in Kozlovski et al.^9^ and avoiding using repeated measures in our context are set to 0.5 and 0, respectively. Number of trees used is 300 with a maximal depth of 5.

From those clinical comparisons (set $B$) and challenges hierarchy (set $W$), STEPS learns a function that maps the $i$^th^ subject's observed feature vector at difficulty level $d$, $x_{i}^{d}$, to a performance score. Thus, STEPS allows for the description of a performance plane per subject by scoring performance in each difficulty level encountered. Next, The STEPS algorithm learns from the stress test features what scale best reconstructs sets $B$ and $W$. Set B imposes a difference in score between subjects that clinically shown to have a difference in their MCR capacities. Set $W$ imposes a difference in scores, within each subject, according to the difficulty load encountered. I.e., we expect that a subject's performance score will be worse in higher difficulty levels.

| **Number of digits** | **Display duration (sec)** | |
| --- | --- | --- |
| 0 | 0 | |
| 1 | 5 | |
| 1 | 3 | |
| 2 | 5 | |
| 2 | 3 | |
| 3 | 5 | |
| 3 | 3 | |
| **Speed relative to pWS** | |  |
| 0% | |  |
| 80% | |  |
| 100% | |  |
| 120% | |  |
| 130% | |  |
| 140% | |  |

Table S2: difficulty levels and their difficulty load hierarchy for the motor domain (left) and cognitive domain (right).

1. **Training a performance scoring model.** A model was trained to score performance in a graded-challenging task, and a score is provided for every challenge level. Given the performance scores, a three-dimensional performance plane that holds the performance scores as a function of the two dimensions of difficulty levels - the cognitive and motor domains was generated for each subject. Performance scores were normalized across all subjects in the training set to have a mean of 0 and SD of 1 (estimated mean and SD were retained to normalize test set's scores).
2. **Constructing a reference performance plane.** A reference plane was constructed as the *median score* in each difficulty level among the control group from the training set. Because the task difficulty increases along both cognitive and motor domains, the plane spans a three-dimensional space representing expected performance across the full range of challenges. This reference plane set the expected performance for a new subject. Similarity to the reference plane represents intact reserve, while a disparity was an indicator of MCR reserve deficit. Similarly, and for validation purposes, we constructed a performance plane for the diagnosed patients group.
3. **Aggregating performance scores to the MCR index**. The aggregated score was calculated as the average difference from one's performance plane to the reference plane. The aggregated scores were then scaled to range between 0-100, with higher values reflecting greater reserve (estimated minimum and maximum values were retained to scale the test set's scores). The resulting score is the *MCR index*^40^.


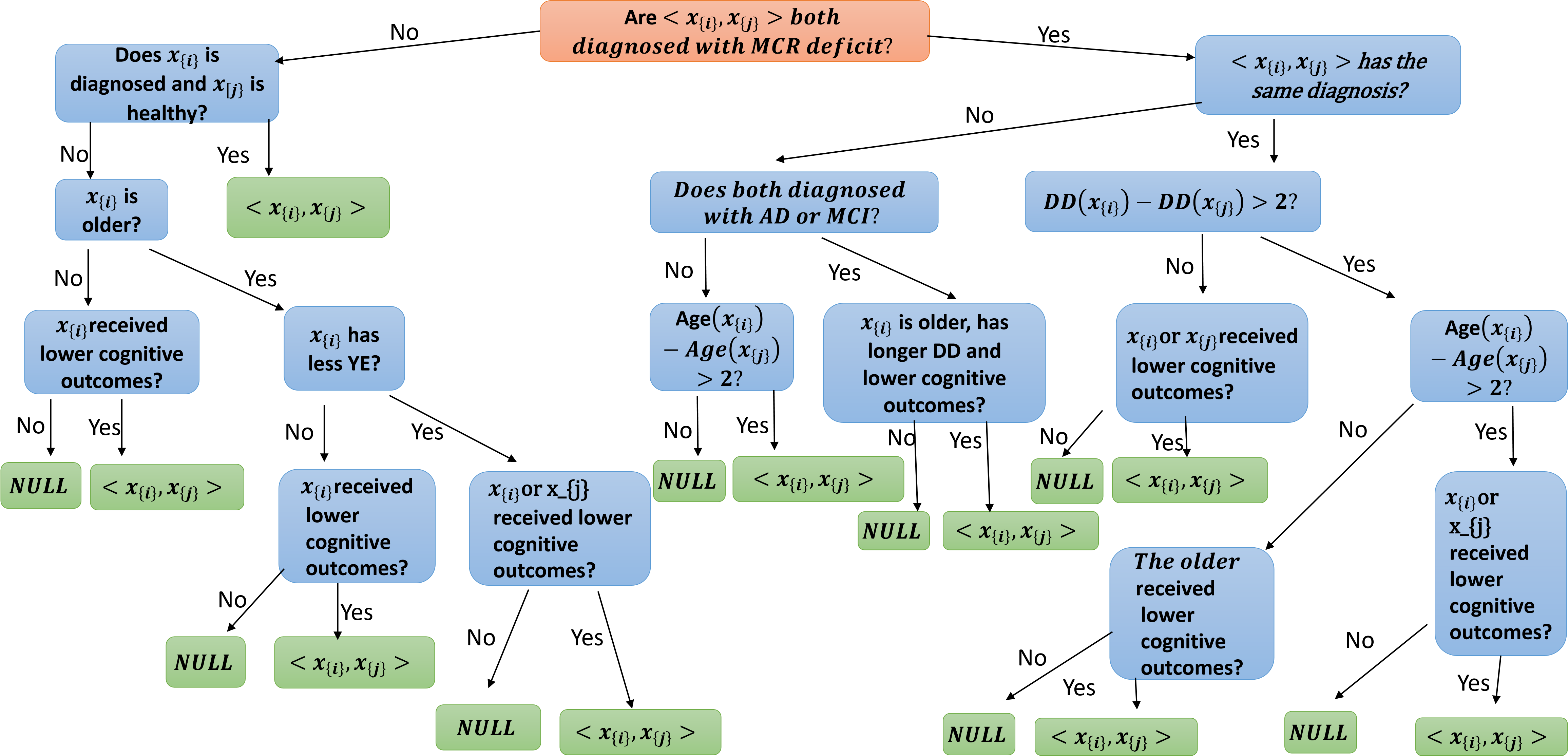


Figure S 1: Ranking scheme according to cognitive domain. AD= Alzheimer’s disease; DD=Disease duration; MCI= Motor cognitive impairment; YE=Years of education.


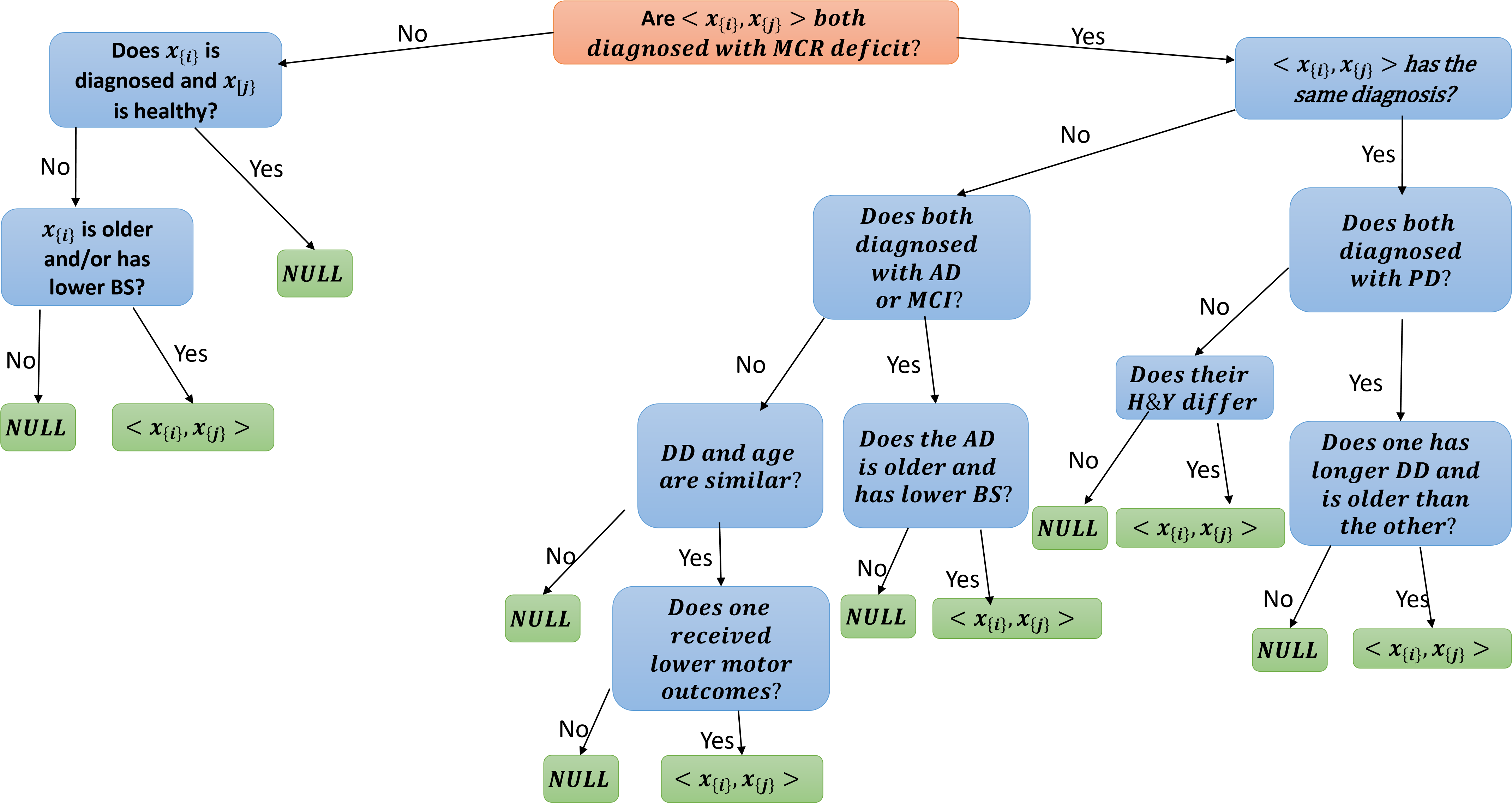


Figure S2: Ranking scheme according to motor domain. AD=Alzheimer’s disease; BS= Base speed; DD=Disease duration; H&Y= Hoen &Yahr; MCI=Motor cognitive impairment; PD=Parkinson’s disease.

**Appendix D**

**Figure S3 Standardized performance scores (Z-scores) distribution for each challenge combination.** The healthy-control group density is in red, and the diagnosed patients are in blue. Rows are the cognitive domain challenge levels (a combination of number of digits and display time of the challenge). Columns are the motor domain challenge levels (Relative speed to preferred walking speed in percentages). Values of "No challenge" represent that no virtual challenge was induced (i.e., a time segment in which the subject only walked on the treadmill). A higher performance score reflects a better result. See Supplementary A and table S2 for further information on the MCR stress test protocol and difficulty levels induced.


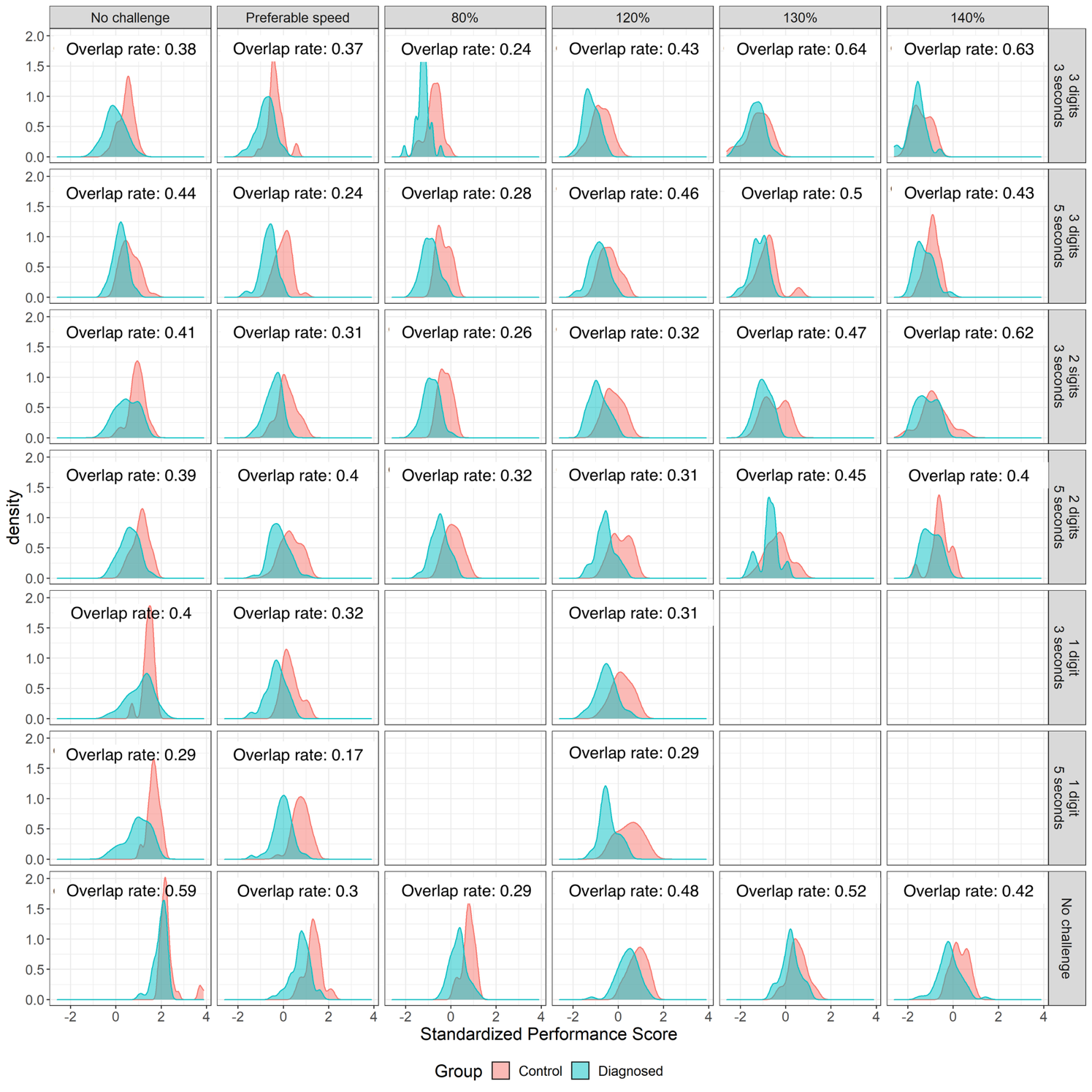


Motor Difficulty Level

Cognitive Difficulty Level

**Appendix E**

**Figure S4 Known groups validity - ROC curves comparison of MCR proxies.** Curves are colored and dashed by measurement. AUC levels as provided in Table 2 are mentioned next to each measurement label in the legends. Grey dashed line is the identity line.


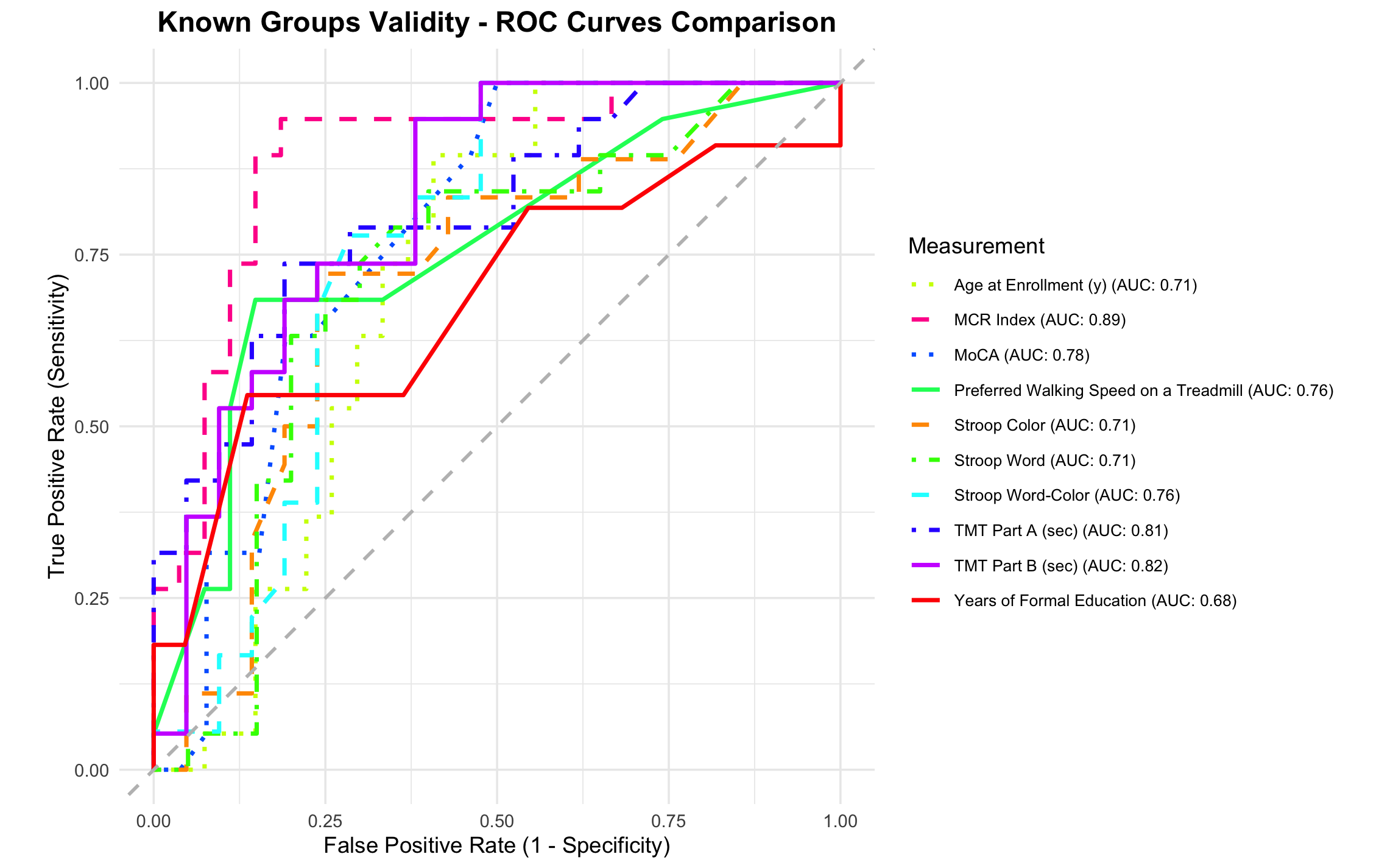


**Appendix F**

**Table S3 – correlations with MRI features**. Spearman's Rho correlations between the MCR index and different MRI volumetric measures, adjusted to total brain volume. Alongside P-values, and the associated BH adjusted P-values to control the false discovery rate.

|  |  | Spearman's Rho coefficient | P-value | BH Adjusted P-value |
| --- | --- | --- | --- | --- |
| MCR Index | Amygdala left | 0.46 | 0.020 | **0.043** |
|  | Amygdala right | 0.31 | 0.136 | 0.175 |
|  | Anterior cingulate right | 0.17 | 0.419 | 0.439 |
|  | Anterior cingulate left | 0.24 | 0.245 | 0.283 |
|  | Caudate left | 0.56 | 0.004 | **0.018** |
|  | Caudate right | 0.68 | 0.000 | **0.004** |
|  | Grey matter | 0.63 | 0.001 | **0.009** |
|  | Hippocampus left | 0.49 | 0.013 | **0.041** |
|  | Hippocampus right | 0.47 | 0.019 | **0.043** |
|  | Inferior frontal gyrus left | 0.47 | 0.019 | **0.043** |
|  | Inferior frontal gyrus right | 0.58 | 0.002 | **0.017** |
|  | Middle frontal gyrus left | 0.44 | 0.027 | 0.055 |
|  | Middle frontal gyrus right | 0.33 | 0.110 | 0.151 |
|  | Pallidum left | -0.06 | 0.776 | 0.776 |
|  | Pallidum right | -0.17 | 0.419 | 0.439 |
|  | Putamen left | 0.40 | 0.048 | 0.081 |
|  | Putamen right | 0.36 | 0.080 | 0.126 |
|  | Superior frontal gyrus left | 0.30 | 0.149 | 0.183 |
|  | Superior frontal gyrus right | 0.35 | 0.091 | 0.133 |
|  | Superior medial frontal gyrus left | 0.40 | 0.048 | 0.081 |
|  | Superior medial frontal gyrus right | 0.49 | 0.012 | **0.041** |
|  | White matter | 0.55 | 0.004 | **0.018** |

Appendix G

**Table S4 - Construct validity’s post-hoc analysis.** We reviewed whether the associations of the MCR index with the MRI data could have been explained solely by age. The table presents the Spearman’s correlation of the MCR index and age at enrollment with the MRI data, alongside a semi-partial correlation of the MCR index with the MRI data after controlling for age on all measures. Colored cells indicate a significant correlation.

| **MRI measure** | **MCR Index** | **Age** | **semi-partial correlation with MCR index** |
| --- | --- | --- | --- |
| Amygdala left | 0.46 | -0.391 | 0.29 |
| Amygdala right | 0.31 | -0.455 | -0.016 |
| Anterior cingulate left | 0.17 | -0.515 | -0.191 |
| Anterior cingulate right | 0.24 | -0.132 | 0.109 |
| Caudate left | 0.56 | -0.421 | 0.411 |
| Caudate right | 0.68 | -0.487 | 0.543 |
| Grey matter | 0.63 | -0.682 | 0.289 |
| Hippocampus left | 0.49 | -0.431 | 0.293 |
| Hippocampus right | 0.47 | -0.466 | 0.221 |
| Inferior frontal gyrus left | 0.47 | -0.592 | 0.094 |
| Inferior frontal gyrus right | 0.58 | -0.522 | 0.356 |
| Middle frontal gyrus left | 0.44 | -0.634 | -0.001 |
| Middle frontal gyrus right | 0.33 | -0.441 | 0.032 |
| Pallidum left | -0.06 | 0.165 | 0.078 |
| Pallidum right | -0.17 | 0.193 | -0.049 |
| Putamen left | 0.4 | -0.311 | 0.268 |
| Putamen right | 0.36 | -0.305 | 0.212 |
| Superior frontal gyrus left | 0.3 | -0.185 | 0.238 |
| Superior frontal gyrus right | 0.35 | -0.522 | -0.028 |
| Superior medial frontal gyrus left | 0.4 | -0.678 | -0.14 |
| Superior medial frontal gyrus right | 0.49 | -0.712 | -0.003 |
| White matter | 0.55 | -0.578 | 0.256 |

**References**

1. Grande, Giulia, et al. "Measuring gait speed to better identify prodromal dementia." *Experimental gerontology* 124 (2019): 110625.‏
2. Cleveland, W. S., E. Grosse, and W. M. Shyu. "Local regression models. Chapter 8 in Statistical models in S (JM Chambers and TJ Hastie eds.), 608 p." Wadsworth & Brooks/Cole, Pacific Grove, CA (1992).‏
3. Schünemann, Holger J., and Gordon H. Guyatt. "Commentary—goodbye M (C) ID! Hello MID, where do you come from?." Health services research 40.2 (2005): 593-597.‏
4. Nasreddine ZS, Phillips NA, Bédirian V, et al. The Montreal Cognitive Assessment, MoCA: a brief screening tool for mild cognitive impairment. J Am Geriatr Soc. 2005;53(4):695-699.
5. Stroop JR. Studies of interference in serial verbal reactions. J Exp Psychol. 1935;18(6):643.
6. Tombaugh TN. Trail Making Test A and B: normative data stratified by age and education. Arch Clin Neuropsychol. 2004;19(2):203-214.
7. Pangman, Verna C., Jeff Sloan, and Lorna Guse. "An examination of psychometric properties of the mini-mental state examination and the standardized mini-mental state examination: implications for clinical practice." Applied Nursing Research 13.4 (2000): 209-213.‏
8. Martinez-Martin P, Rodriguez-Blazquez C, Alvarez-Sanchez M, et al. Expanded and independent validation of the Movement Disorder Society--Unified Parkinson’s disease rating scale (MDS-UPDRS). J Neurol. 2013;260(1):228-236.
9. Kozlovski, Tal, et al. "A novel performance scoring quantification framework for stress test set-ups." Plos one 18.4 (2023): e0284083.‏
